# Experimental and CFD analysis of fluid flow through nanofiber filter media

**DOI:** 10.1038/s41598-024-67066-x

**Published:** 2024-07-12

**Authors:** Mehdi Azimian, Matin Naderi, Parham Soltani, Liping Cheng, Keivan Naderi, Sven Linden, Andreas Wiegmann

**Affiliations:** 1 Math2Market GmbH, Kaiserslautern, Germany; 2 Department of Textile Engineering, Isfahan University of Technology, Isfahan, 84156-83111 Iran

**Keywords:** Air filtration, Nanofiber filter medium, CFD, Slip flow, Permeability, Filtration performance, Chemical engineering, Computational methods, Fluid dynamics

## Abstract

This work presents a novel approach to investigating the slip effect in nanofiber filter media. Electrospun nanofiber media with high efficiency and low pressure drop were produced at different concentrations and durations. The surface and cross-sectional morphology of nanofiber media were studied using FE-SEM. Fiber orientation and diameter distributions were also examined. The 3D virtual nanofiber media was modeled using this information along with the experimentally measured porosity and thickness of the media. The effect of the slip phenomenon in nanofiber media was studied numerically, and the results were compared to experimental data. Excellent agreements were found between the measured and simulation results. Additionally, filtration simulations considering aerosols injected with airflow through the nanofibrous filter media were conducted by considering the slip effect, and the effect of filter structure on filtration performance (removal efficiency and pressure drop) was investigated.

## Introduction

Nanofibrous filter media in buildings and industries are of utmost importance for their ability to efficiently capture and remove microscopic particles, such as allergens and pollutants, thereby improving air quality and ensuring a healthier and more conducive environment^1–4^. In addition, the growing need to filter fine particles and aerosols containing viruses like COVID-19 has boosted the use of nanofibers considerably more appealing^5–8^. Flow behavior is the most important parameter influencing the engineering design of such structures in these applications. To fully comprehend fluid flow in these structures, the influence of internal structure at the micro- and nanoscales must be investigated.

Studies on the investigation of fluid flow in fibrous media include experimental, analytical, and numerical computer simulation-based methods. A detailed review of these studies can be found in^9–11^. Although some of the well-established models for the prediction of fluid flow behavior in fibrous structures are based on experimental data^12–14^, because of the macroscopic nature of these methods, the influence of fibrous media structure on fluid flow at the micro- and nanoscale cannot be fully investigated. Therefore, as alternative methods, analytical solutions^15–19^ and numerical approaches^20–25^ have been established to examine the influences of the microstructure on fluid flow behavior. However, these models cannot be used directly to predict fluid behavior in nanofibrous structures and, generally, underpredict the fluid velocity in nanofibrous media. This is because, in these studies, the no-slip boundary condition (NSBC) is assumed to exist on the surfaces of the fibers, which renders the aforementioned models inaccurate. It has been well-established that when the gas mean free path is of the same order as the fiber diameter, the continuum assumption is no longer valid and a velocity slip may take place^26–30^.

In the context of nanofibrous filter media, finding the optimized characteristics of nanofibrous structures such as thickness, porosity, fiber diameter distribution, and 3D fiber orientation distribution through experimental procedures is quite expensive and a big challenge. Additionally, various physical phenomena, including particle adhesion effects and *Brownian* motion, make modeling the fluid flow through the nanofibrous structures very difficult^31^. Such optimization issues can be resolved by using novel GeoDict modeling and simulation capabilities. In this software, the 3D fibrous structure is converted to voxel data with staircase boundaries at the surfaces of the fibers. Imposing wall shear stress on such staircase surfaces is an enormous challenge. Additionally, the simulation of the fibrous filter medium and filtration process is carried out by resolving the smallest scales of the medium. Although this method enjoys the advantages of keeping the number of model parameters and hierarchy minimal, two major problems occur in the nano-regime. First, the simulated structure becomes extremely large, and special algorithms should, therefore, be developed to resolve this difficulty. Secondly, models capable of describing the slip flow phenomena should be established to simulate fluid flow and the movement of particles. Therefore, in this work, a description of a developed modeling approach for slip flow simulation on voxelized nanofibrous structures is presented. Three different nanofibrous filter media are produced, and their air permeabilities are measured experimentally. The 3D virtual structure of media is modeled, and fluid flow behavior is investigated using the developed model. Finally, the computational results are compared and validated with the experimental data.

## Implementation of slip-length for nanofibers

To simulate the filtration process, the flow field needs to be solved. For creeping flow, it is defined by the steady-state *Stokes* equations (momentum balance and mass conservation) for incompressible *Newtonian* fluid as follows:

1 $$ - \mu \Delta \vec{u} + \nabla p = 0 \left( {momentum\; balance} \right) $$

$$ \nabla \cdot \vec{u} = 0 \left( {mass \;conservation} \right) $$

where $\mu$ denotes air viscosity, $\overrightarrow{u}$ represents the periodic velocity and is zero on fiber surfaces, $p$ is the air pressure, and ${P}_{in}={P}_{out}+c$, where $c$ is the constant pressure drop applied on the filter medium.

In previous studies, the implementation of slip-flow boundary conditions (SBC) in voxel-based flow solvers could not be properly achieved. Generally, in these works, two methods were used as workarounds: the equivalent shrunk fibers and the equivalent permeable fibers. In the former, the fibers are shrunk to permit fluid flow on the fibers' surface voxels, while in the latter method, the fibers are permeable to allow fluid flow through the fibers. In these methods, the structure should be of very high resolution, and large computational efforts are required to find the appropriate hypothetical permeability values^32–35^.

In the proposed approach in this study, it is assumed that at the solid wall, the slip velocity is proportional to the shear stress, and the slip velocity expression is reformulated for a locally quadratic velocity profile rather than the standard linear profile. This approach makes it possible to directly simulate the slip flow.

To this end, the finite difference technique proposed by Wiegmann^36^ is used, and the velocities are interpolated from the cell faces to obtain a continuous velocity field, which is required for particle tracking. For nanofibers, SBC with a slip length of $\delta$ is applied as follows:

2 $$ \vec{n} \cdot \vec{u} = 0 \;{\text{on}}\; \Gamma \left( {{\text{no}} \;{\text{flow}}\; {\text{in}} {\text{to}} \;{\text{fibers}}} \right) $$

3 $$ \vec{t} \cdot \vec{u} = - \delta \vec{n} \cdot \nabla \left( {\vec{u} \cdot \vec{t}} \right) \;{\text{on}}\; \Gamma \left( {{\text{slip }}\;{\text{flow}}\;{\text{ along}}\;{\text{ fibers}}} \right) $$

where $\overrightarrow{n}$ denotes the normal direction to the fiber axis, and $\overrightarrow{t}$ represents any tangential direction with $\overrightarrow{t} \cdot \overrightarrow{n}=0$.

Finding the tangential directions of the voxelized structure of randomly placed fibers is one of the main challenges and restrictions of this method. On cubic cells, it was previously possible to only resolve the axis-parallel tangents, which restricted the capability of computing cases with large slip lengths.

**Figure 1:**
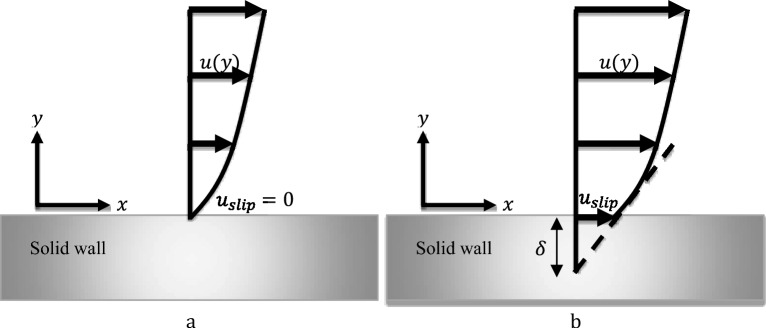
Schematic diagram of (**a**) NSBC and (**b**) SBC on a solid surface.

The gray fiber in Fig. 1 serves as a solid wall for a straight channel. To determine the velocity along with the fiber (${u}_{slip}$), the coordinate system is set as $x$ along the fiber axis and $y$ normal to the fiber surface. For the no-slip flow regime ($\delta =0$), fluid velocity gradually decreases to zero at the solid–liquid interface. For the slip flow regime, the slip length $\delta$ can be interpreted as the hypothetical distance below the solid wall-liquid interface where velocity extrapolates to zero. In this condition, the relative velocity is non-zero and the degree of slip is manifested by the $\delta$ as follows:

4 $$ u_{slip} = \delta \frac{\partial u}{{\partial y}} \;at\; y = 0 $$

For example, by taking the first-order discretization, the velocity at the surface, $u_{0}$, satisfies:

5 $$ u_{0} = \frac{{u_{i} + u_{i - 1} }}{2} $$

**Figure 2:**
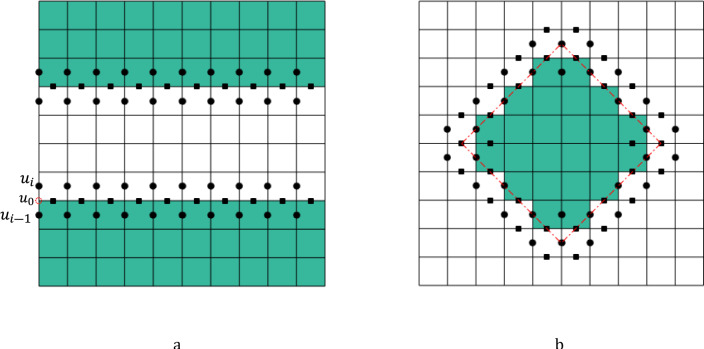
The surface velocity components for a surface (**a**) parallel to coordinate axes and (**b**) inclined to coordinate axes.

Substituting the velocity at the cell above the surface, $u_{i}$, and the slip velocity $u_{0}$ into Eq. 4 gives:

6 $$ u_{0} = \delta \frac{{u_{i} - u_{0} }}{\frac{h}{2}} $$

where ${u}_{i}$ and ${u}_{i-1}$ denote the velocity in the flow and solid, respectively (see Fig. 2a for an example of locations of ${u}_{i}$ and ${u}_{i-1}$), and $h$ is the length of the grid cell. The value for ${u}_{i-1}$ can be then expressed as:

7 $$ u_{i - 1} = \frac{{\delta - \frac{h}{2}}}{{\delta + \frac{h}{2}}}u_{i} $$

In the staggered grid, the velocity components are located differently. In the 2D examples shown in Fig. 2, the circular dots denote the locations of the velocity component in the left–right (horizontal) direction, and the squared dots mean those in the top–bottom (vertical) direction. Figure 2a shows the locations of velocity components for a parallel channel, and Fig. 2b indicates an example of a surface inclined to the coordinate axes.

**Figure 3:**
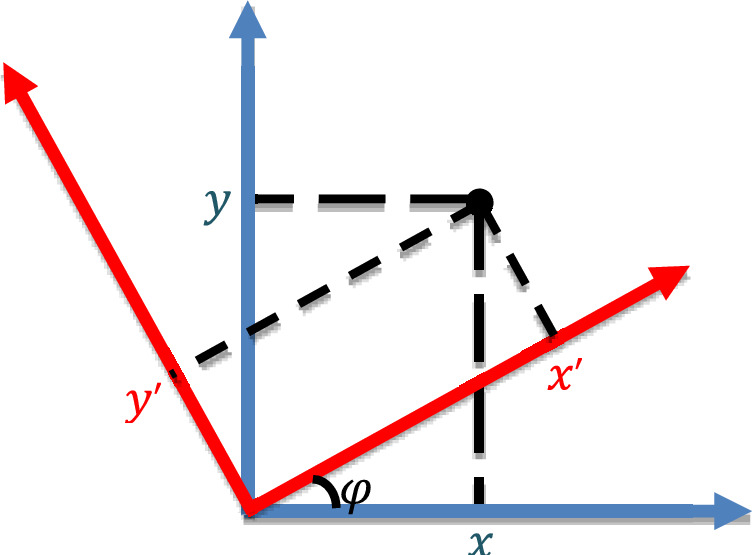
Coordinate system rotation.

When the slip surface is inclined to coordinate axes (red lines in Fig. 2b), the problem becomes more challenging, and one needs to derive the expressions for different velocity components in Eq. 4. Assuming the angle between the slip surface and $x$-axis is $\theta$ (Fig. 3), for the 2D case, Eq. 4 holds in the coordinate system $\left( {x^{\prime},y^{\prime}} \right)$:

8 $$ u^{\prime} = \delta \frac{{\partial u^{\prime}}}{{\partial y^{\prime}}}\; {\text{and}}\; v^{\prime} = 0 $$

$u^{\prime}$ and $v^{\prime}$ denote the velocity components in the $x^{\prime}$ and $y^{\prime}$ directions. Coordinate transformation (Fig. 3) can be done. The velocity components in the $(x,y)$ are defined as follows:

9 $$ u = u^{\prime}\cos \varphi \;{\text{and}}\;v = u^{\prime}\sin \varphi $$

Substituting Eq. 9 into Eq. 8 gives:

10 $$ \left. u \right|_{x = 0, y = 0} = \frac{\delta }{\cos \varphi } \left. {\frac{\partial u}{{\partial x}}} \right|_{x = 0, y = 0} = \frac{\delta }{\sin \varphi } \left. {\frac{\partial u}{{\partial y}}} \right|_{x = 0, y = 0} $$

11 $$ \left. v \right|_{x = 0, y = 0} = \frac{\delta }{\cos \varphi }\left. { \frac{\partial v}{{\partial x}}} \right|_{x = 0, y = 0} = \frac{\delta }{\sin \varphi }\left. { \frac{\partial v}{{\partial y}}} \right|_{x = 0, y = 0} $$

By discretizing and implementing Eqs. 10 and 11, the slip flow can be solved for the voxelized structure depicted in Fig. 2b. For the 2D case, the planar Poiseuille flow can be used to validate because an analytic solution can be easily obtained.

**Figure 4:**
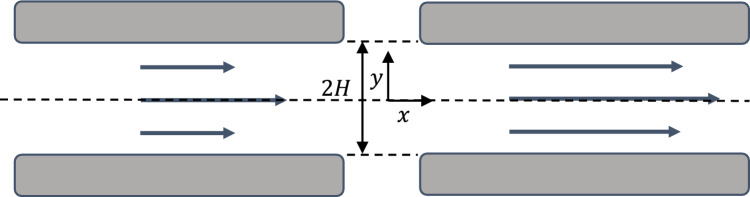
NSBC and SBC for planar Poiseuille flow.

Suppose the channel width is 2H (Fig. 4), the momentum equation (Eq. 1) is reduced to:

12 $$ \mu \frac{{\partial^{2} u}}{{\partial y^{2} }} = \frac{{{\text{d}}P}}{{{\text{d}}x}} $$

where $\frac{{{\text{d}}P}}{{{\text{d}}x}}$ is a constant.

For NSBC:

13 $$ \left. u \right|_{ y = H} = \left. u \right|_{ y = - H} = 0 $$

and

14 $$ \left. {\frac{du}{{dy}}} \right|_{y = 0} = 0 $$

For SBC, Eq. 14 still holds but Eq. 13 becomes:

15 $$ \left. u \right|_{ y = H} = \left. u \right|_{ y = - H} = - \delta \left. {\frac{{{\text{d}}u}}{{{\text{d}}y}}} \right|_{ y = H} = \delta \left. {\frac{{{\text{d}}u}}{{{\text{d}}y}}} \right|_{ y = - H} $$

Solving Eqs. 12, 13, and 14 to obtain the solution for the no-slip flow as:

16 $$ u = - \frac{1}{\mu }\frac{{{\text{d}}P}}{{{\text{d}}x}}\left( {\frac{{H^{2} - y^{2} }}{2}} \right) $$

Solving Eqs. 12, 14, and 15 to obtain the solution for the slip flow as:

17 $$ u = - \frac{1}{\mu }\frac{{{\text{d}}P}}{{{\text{d}}x}}\left( {\frac{{H^{2} - y^{2} + 2H\delta }}{2}} \right) $$

**Figure 5:**
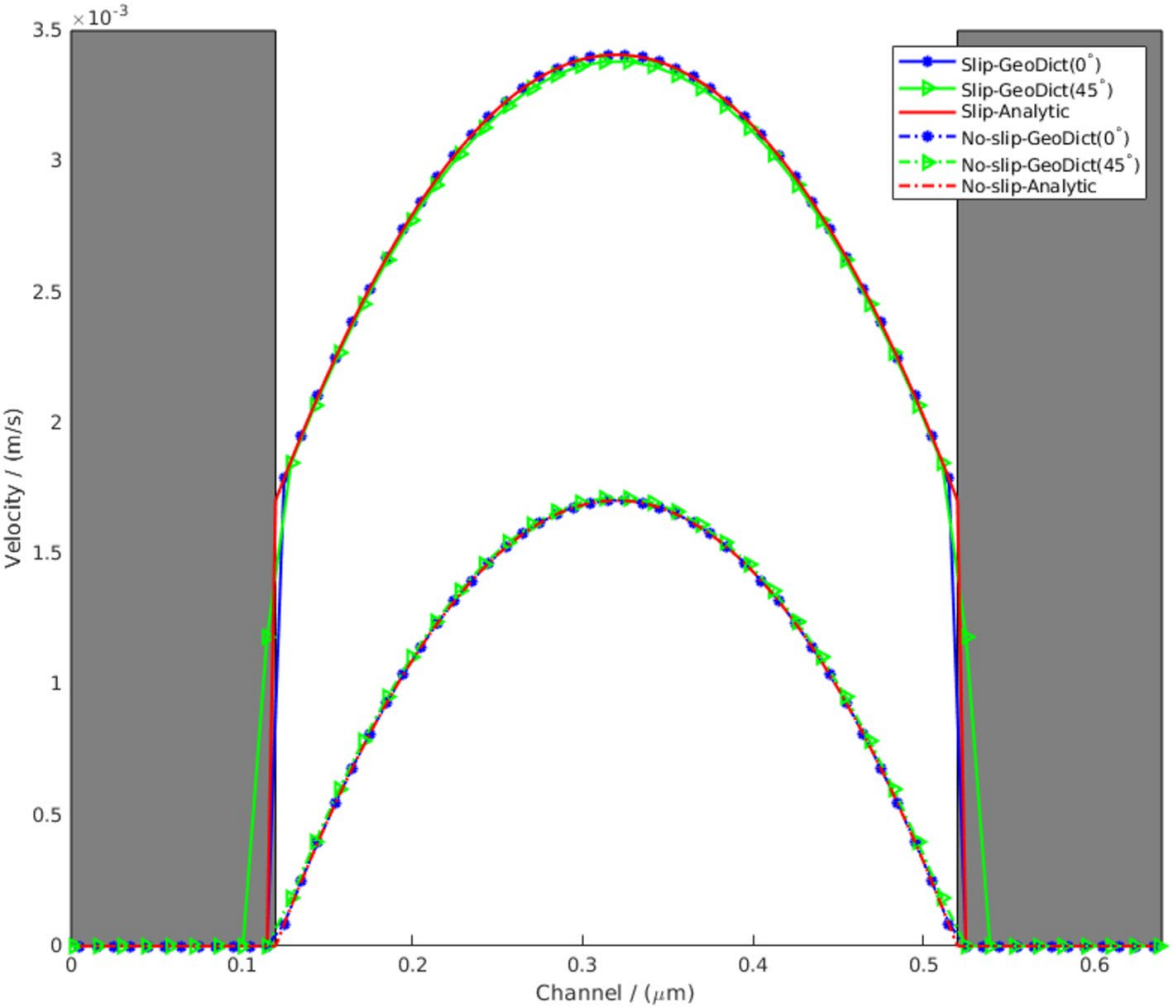
Comparison of the simulated result and analytic solutions for planar Poiseuille flow.

**Figure 6:**
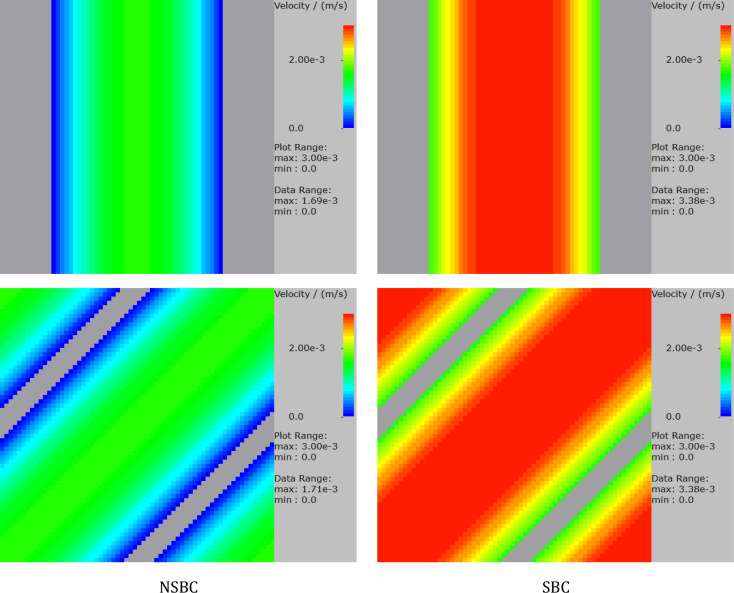
Comparison of the NSBC and SBC for different angles for planar Poiseuille flow.

The planar Poiseuille flow was solved in a channel of 400 nm at different angles to validate the algorithm. The air viscosity is 1.834e−5 kg/(m s) and the pressure drop in the flow direction is 1 Pa. The voxel length used in this example is 10 nm, and the length of the channel is 64 voxels. A slip length of 70 nm which is equal to the air mean free path length is used. The comparison of the results for the NSBC and SBC is given in Fig. 5. Because in the simulation the velocity component in flow direction does not have a variable on the interface between solid and fluid voxel (Fig. 2), the values, therefore, cannot be directly compared. The values in the gray boxes, which are solid, are only numerical and are set to zero. Therefore, we only need to concern with the values in the fluid region. As observed, the results point to a very good agreement with the analytical solutions. The visualizations of the velocity distributions are given in Fig. 6.

**Figure 7:**
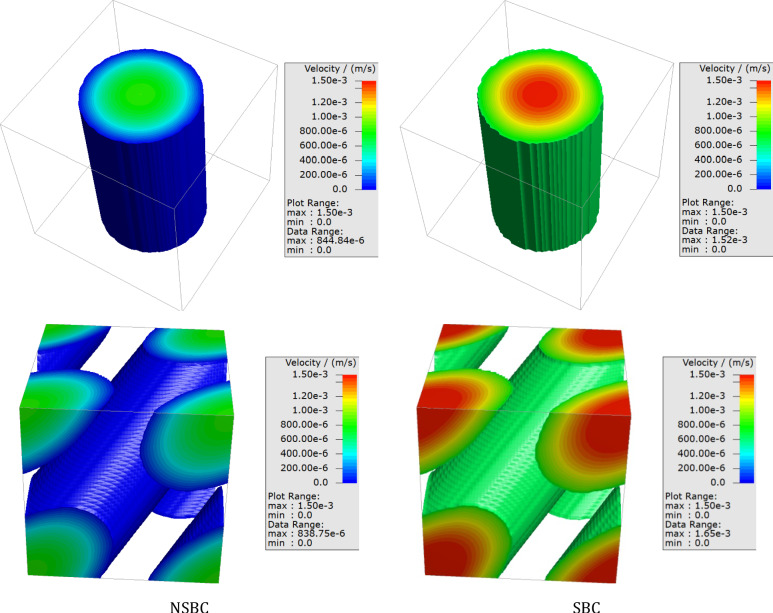
Comparison of the NSBC and SBC for different angles for Couette–Poiseuille flow.

For the 3D case, the Couette–Poiseuille flow was solved in a pipe, and the results are presented in Fig. 7. As is seen, accurate results are achieved for different angles between the pipe and the coordinate axes. The velocity profiles in the channel are the same regardless of whether the channel is perpendicular to a coordinate axis or not when the same boundary condition is applied.

The main challenge in the simulation of the slip flow in a nanofibrous structure is to find the angle of each nanofiber. To this end, different image processing algorithms were used to automatically calculate the angle for each nanofiber. For more details, please see the previous works of the authors^37,38^.

For computing the flow field through the medium, the *Navier–Stokes* equations and the *continuity* equation are used to express the conservation of momentum and the conservation of mass as follows:

18 $$ - \mu \Delta \vec{u} + \left( {\rho \vec{u} \cdot \nabla } \right)\vec{u} + \nabla p = \vec{f}\left( {{\text{conservation }}\;{\text{of}}\;{\text{ momentum}}} \right) $$

19 $$ \nabla \cdot \vec{u} = 0\left( {{\text{conservation}}\;{\text{of}}\;{\text{mass}}} \right) $$

where $f$ denotes the force density field and $\rho$ is the fluid density.

On the surfaces of fibers, the SBC was applied as explained in Eq. 2. The fluid properties of air at given working conditions should be given. Periodic boundary conditions are used when a periodic piece of media structure is taken; otherwise, symmetric boundary conditions should be considered. The voxel size, in the range of 20–50 nm, was chosen small enough to resolve the diameter of nanofibers and big enough to prevent memory shortage caused by the huge domain size.

The computed velocities for the SBC are higher than those of the NSBC for a given pressure drop. Conversely, for the same mass flux, the pressure drop is lower for the SBC as compared with the NSBC.

## Experimental

### Electrospinning

Polyacrylonitrile (PAN) in powder form was used without further purification (average molecular weight of 140,000). PAN was dissolved in distilled dimethylformamide to achieve solutions of 10 and 13 wt%. The solutions were stirred magnetically for 20 h at 65 °C to prepare homogeneous solutions. The obtained solutions were injected into a syringe equipped with a stainless-steel needle having a 22 gauge needle. The process was conducted at a temperature of 23 °C, voltage of 16 kV, solution feed rate of 1.1 ml/h, and different spinning times. The distance between the spinneret and the grounded collector, which was covered with stainless steel mesh was 15 cm. Three nanofibrous filter media were produced.

### Air permeability

The nanofibrous samples were kept under vacuum for 24 h after collection to ensure that the solvent was fully evaporated. The air permeability of samples was determined following ASTM-D737 via the Shirley apparatus. The apparatus measures the air volume flow rate (cm^3^/s) passing through 5 cm^2^ of the specimen at a given pressure drop (100 Pa). At least 15 specimens were tested for each sample.

### Morphological analysis

**Figure 8:**
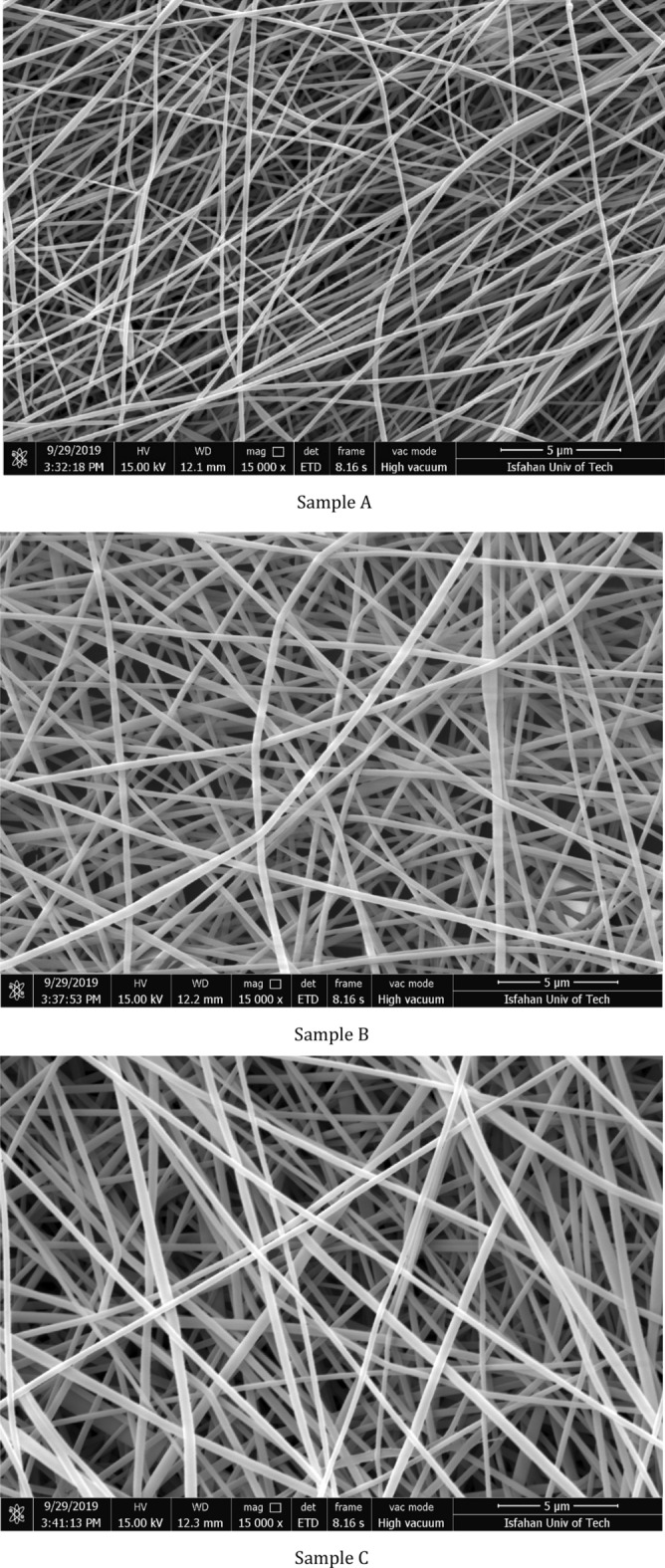
FE-SEM images of nanofibrous filter media.

**Figure 9:**
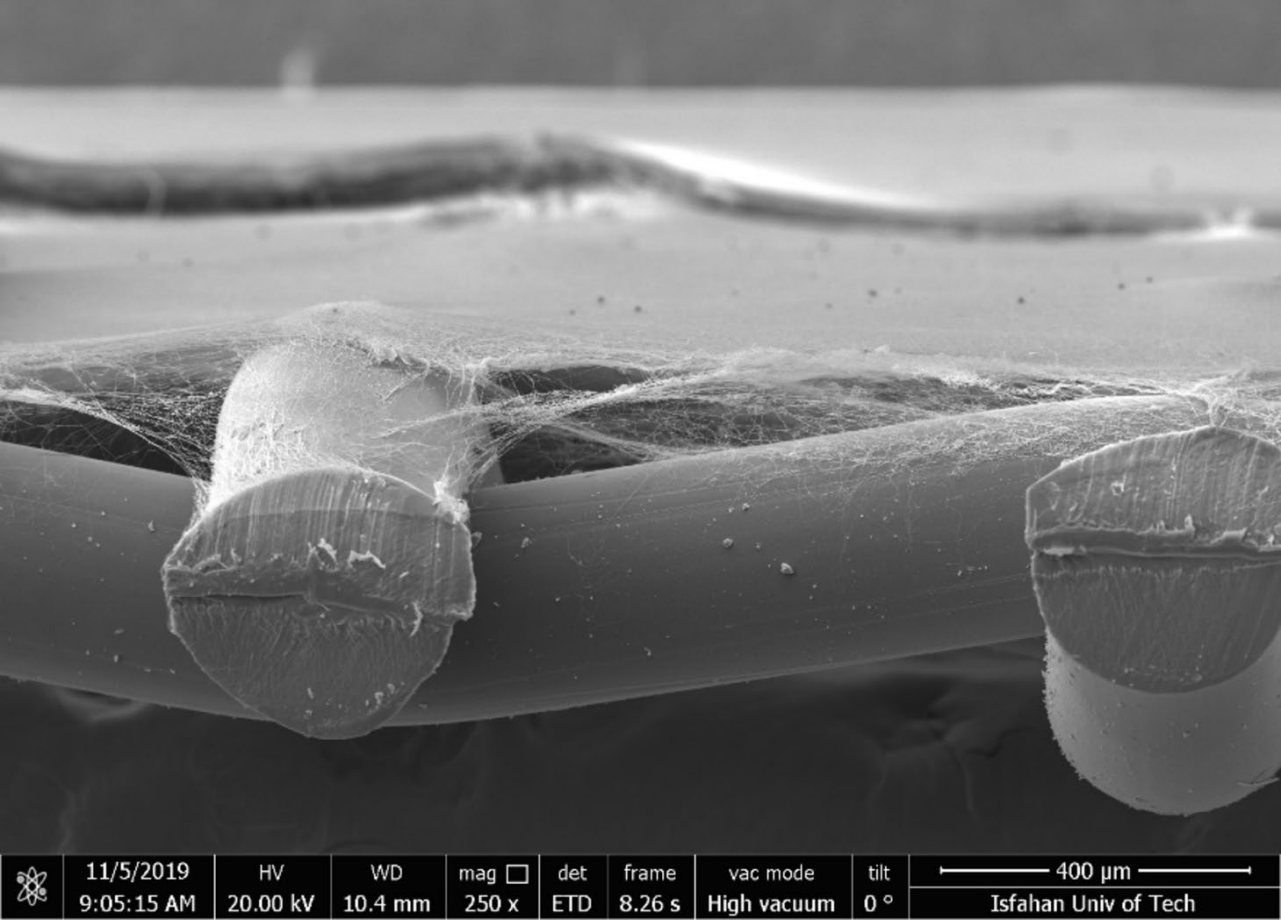
Cross-sectional FE-SEM image of nanofibrous filter medium A on the support mesh.

The surface and cross-sectional morphology of nanofiber media were studied using FE-SEM (MIRA3 TESCAN, Czech). Before the observation, the samples were gold-sputtered using a sputter coater (SCD005, Bal-Tec, Germany) for 120 s. Figure 8 shows the FE-SEM images of samples A–C. The FE-SEM image taken from the cross-section of sample A showing the nanofibrous structure on top of a support mesh is also presented in Fig. 9. The FE-SEM images have a resolution of 18 nm. This was approved by considering the length scale shown at the bottom of the image and the total number of voxels along the scale line.

The thickness of nanofibrous media was measured from the cross-sectional FE-SEM images. The results are given in Table 1. The basis weight of the nanofiber filter media was measured by a balance (AW120, Shimadzu, Tokyo, Japan) with an accuracy of ± 0.0001 g. The basis weight and thickness of the nanofibrous media were used to calculate the porosity of the media.

**Table 1:** Average thickness and fiber diameter of nanofibrous filter media.

Sample	Concentration (wt%)	Fiber diameter (µm)	Thickness (µm)
A	10	0.211 (0.054)	9.66 (1.84)*
B	13	0.301 (0.081)	41.92 (3.05)
C	13	0.352 (0.115)	61.25 (6.10)

*Values in the parentheses indicate the standard deviations.

**Figure 10:**
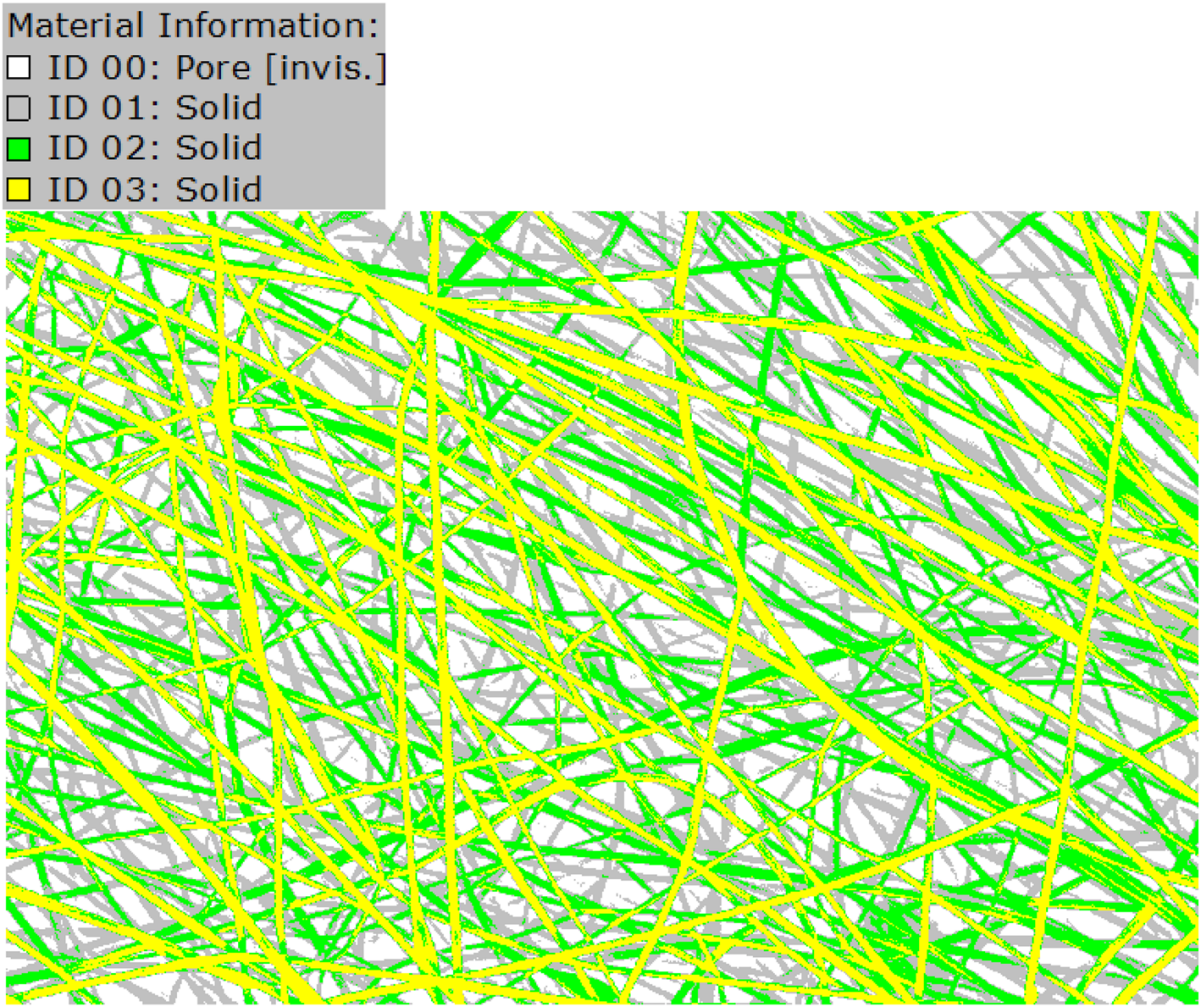
Segmented FE-SEM image using the Otsu method with three threshold values for sample A.

The FE-SEM images were imported into GeoDict and segmented to identify the fibers and voids and separate material IDs. The Otsu method was employed to automatically perform clustering-based image thresholding and transform the grayscale images into binary ones^39^. For the imported FE-SEM images, three threshold values were used to identify the fibers in the front, and background layers, as shown in Fig. 10. As can be observed, the yellow fibers (ID 03) are in the front layer, and the gray ones (ID 01) are in the rear layers. This segmented image can be used for further processing to investigate the morphological features of the samples, such as fiber diameter size distribution and fiber orientation distribution.

The algorithm to estimate fiber diameters works as follows: At the start, each fiber voxel is assigned the value of the diameter of the largest sphere that fits into the fiber in this position. Then each fiber voxel is checked whether it lies inside a larger sphere compared to the diameter of the sphere already assigned to the voxel under consideration. This explanation is valid when dealing with 3D images, e.g., from *µ*CT-scans. When dealing with 2D FE-SEM images, instead of spheres, circles will be fitted into fibers. The results are given in Table 1.

To compute fiber orientations, the Star Length Distribution (SLD) algorithm was used, which works based on a per-voxel analysis. The algorithm evaluates chord lengths through each voxel for a pre-determined set of directions for each voxel. The details of the algorithms are explained in the authors’ previous work^38^.

**Figure 11:**
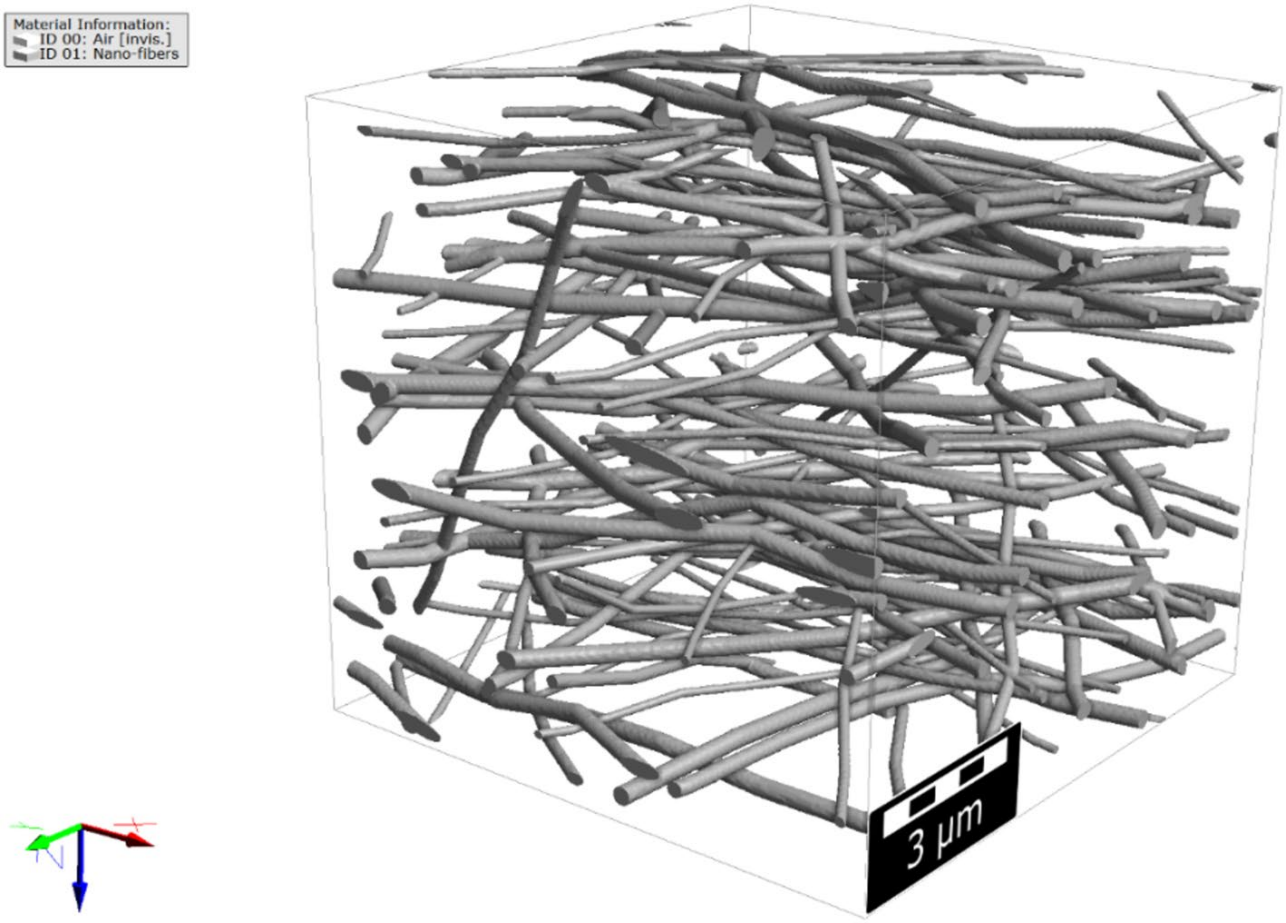
Generated nanofibrous media based on the FE-SEM image for sample A.

The porosity and thickness of the nanofiber filter media, the computed fiber diameter distribution, and the 2D fiber orientation distributions were then used as input parameters to generate the digital 3D structures of the three fibrous media, as shown, e.g., for sample A in Fig. 11. The fibers cross-section was considered circular, and the fibers were modeled as curved fibers similar to what was observed from the FE-SEM images. On the simulated structure, different analyses can be run to find out, e.g., the pore size distribution, percolation path, and bubble point. Moreover, flow simulation was carried out through the medium with the flow in $z$-direction, based on the coordinates shown in Fig. 11.

## Results and discussion

**Figure 12:**
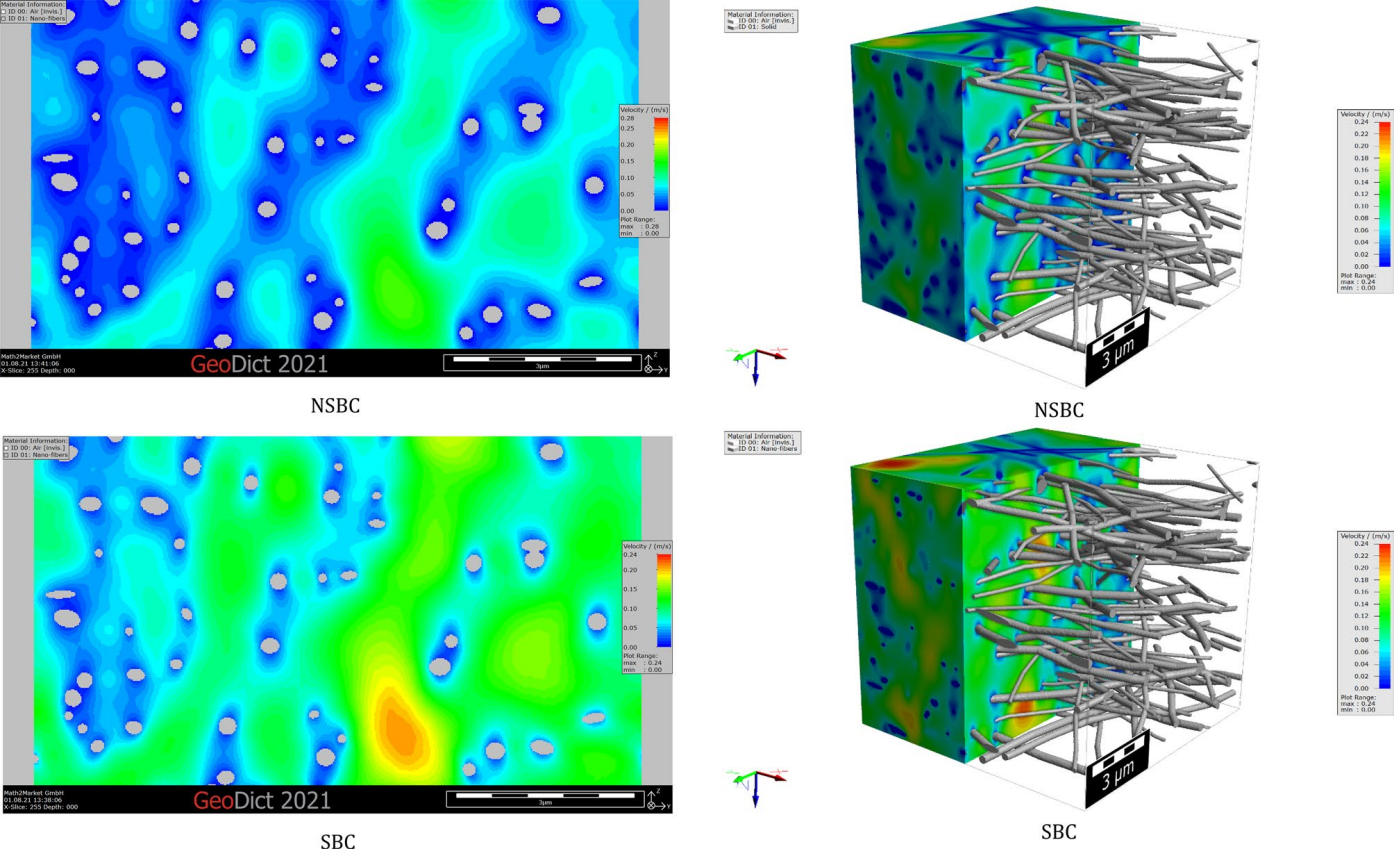
The velocity distributions on a 2D slice and through the 3D simulated filter medium A.

**Figure 13:**
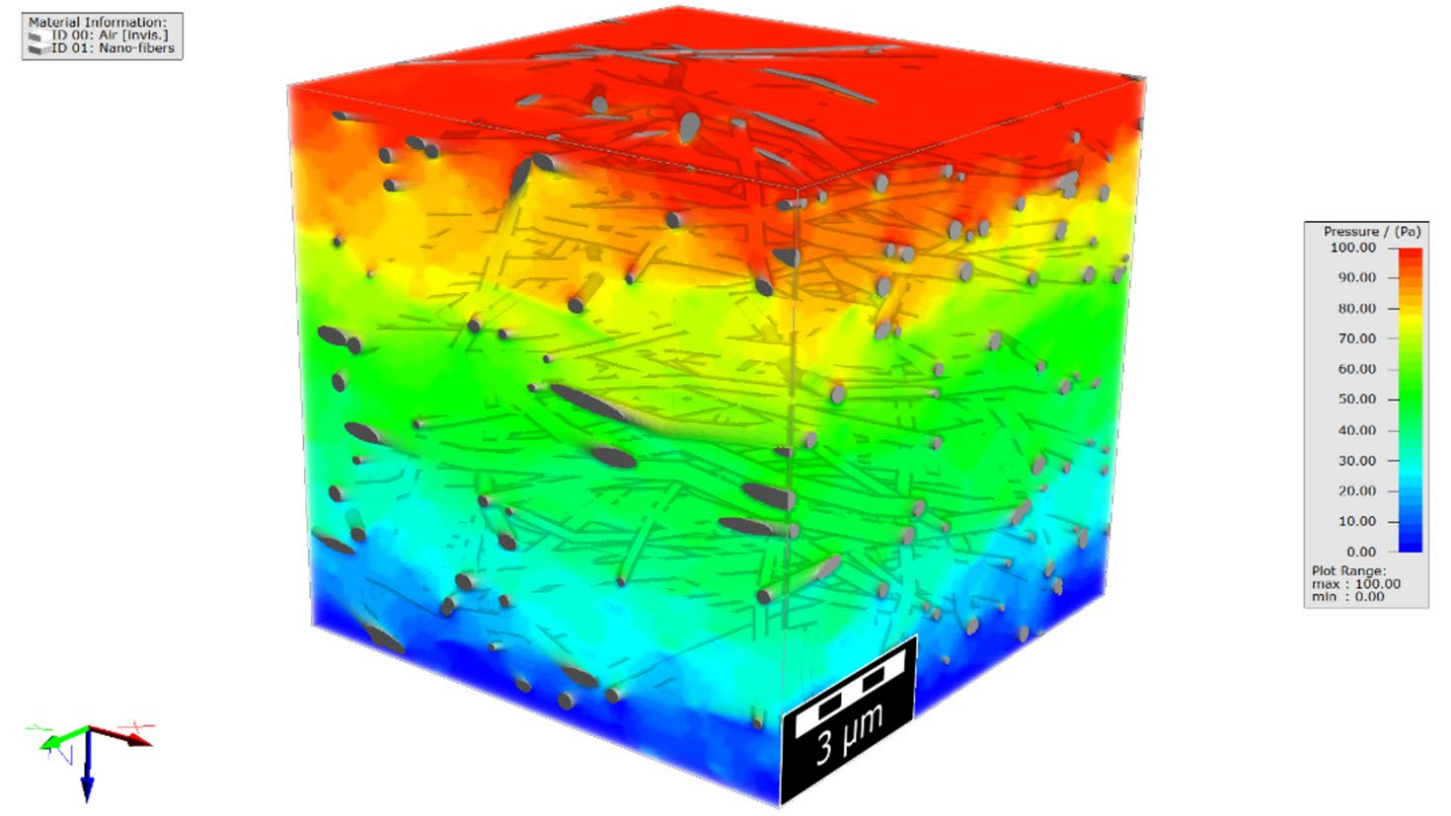
The pressure distribution through the 3D simulated filter medium A.

The comparison of the simulation results in the slip flow regime to those of no-slip flow shows that for a given pressure drop, the computed velocities are considerably underestimated when the NSBC is considered. Figure 12 shows the flow field distribution on a 2D slice of filter medium A for NSBC and SBC. The velocity distributions through the digital 3D filter medium A, by NSBC and SBC, are also shown in this figure. The simulations are conducted considering a constant pressure drop of 100 Pa through the media, as was also considered in the experimental measurement of air permeability through the nanofibrous filter media. The pressure distribution through sample A is shown in Fig. 13.

The structural properties of samples A, B, and C and the experimentally and numerically calculated permeabilities are given and compared in Table 2. As is observed, the agreement between the experimental data and simulations is good. The deviations may be ascribed to the fact that accurate measurement of nanofibrous structures' thickness and porosity is difficult. Moreover, experimental measurements of permeability are subject to experimental error and variability due to factors such as instrumentation, sample preparation, and environmental conditions. Additionally, in simulation, fibers are considered solid rods and are not displaced or deformed, whereas in reality they can be deformed due to the flow of air. It is also noticed that for samples of the same porosity (samples A and B), air permeability reduces with the increase in nanofiber medium thickness. As the thickness increases, the path that a fluid must follow through the material also becomes longer. This longer path results in increased resistance to flow and decreased air permeability.

**Table 2:** The properties of nanofibrous filter media.

Sample	Fiber diameter	Thickness	Porosity	Air permeability: experiment	Air permeability: simulation
	(nm)	(µm)	(%)	(l/m^2^ s)	(l/m^2^ s)
A	211	9.66	93	96.9	90.3
B	301	41.92	93	44.1	39.2
C	372	61.25	97	58.6	54.9

It is also noticed that despite the larger thickness of sample C, the air permeability is higher than that of sample B, which is due to the higher porosity of sample C. When the porosity increases, more void spaces become available for air to flow through, thus providing less resistance to airflow. This suggests that in cases where both porosity and thickness are altered, the changes in porosity can have a more significant effect on permeability.

**Figure 14:**
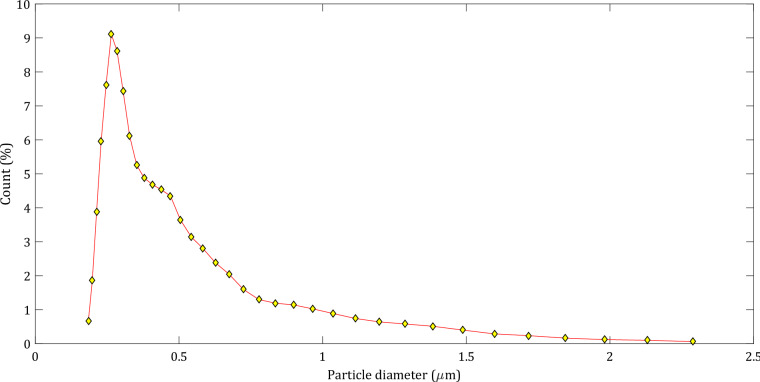
D EHS aerosol size distribution injected with airflow.

To further elucidate the filtration performance of the samples, filtration simulations considering aerosols injected with airflow through the filter media were conducted. The mean flow velocity was considered to be 5.3 cm/s. The flow under this condition was computed by solving *Stokes* equations and considering SBC. DEHS aerosols with a size distribution of 0.019–2.3 µm (Fig. 14), and a density of 910 kg/m^3^, were considered for the filtration simulations through samples A, B, and C. For both the flow and filtration simulations, periodic boundary conditions in flow and tangential directions were considered. The choices made for the boundary conditions in tangential directions also determine what happens when a particle reaches the domain boundary in one of the tangential directions. With periodic boundary conditions, the particle will leave the domain and reappear on the opposite side.

The flow through the filter media is computed with the LIR flow solver^40^. The estimated flow field allows for the tracking of particles. To track particles, an ordinary differential equation that takes into account phenomena such as diffusive motion, electrostatic attraction between particles and the filter surface, and friction with the fluid is solved. When particles touch the filter surface, based on the selection of an adhesion model, it is decided whether the particle sticks to the surface or bounces, slides, and continues to move further.

**Figure 15:**
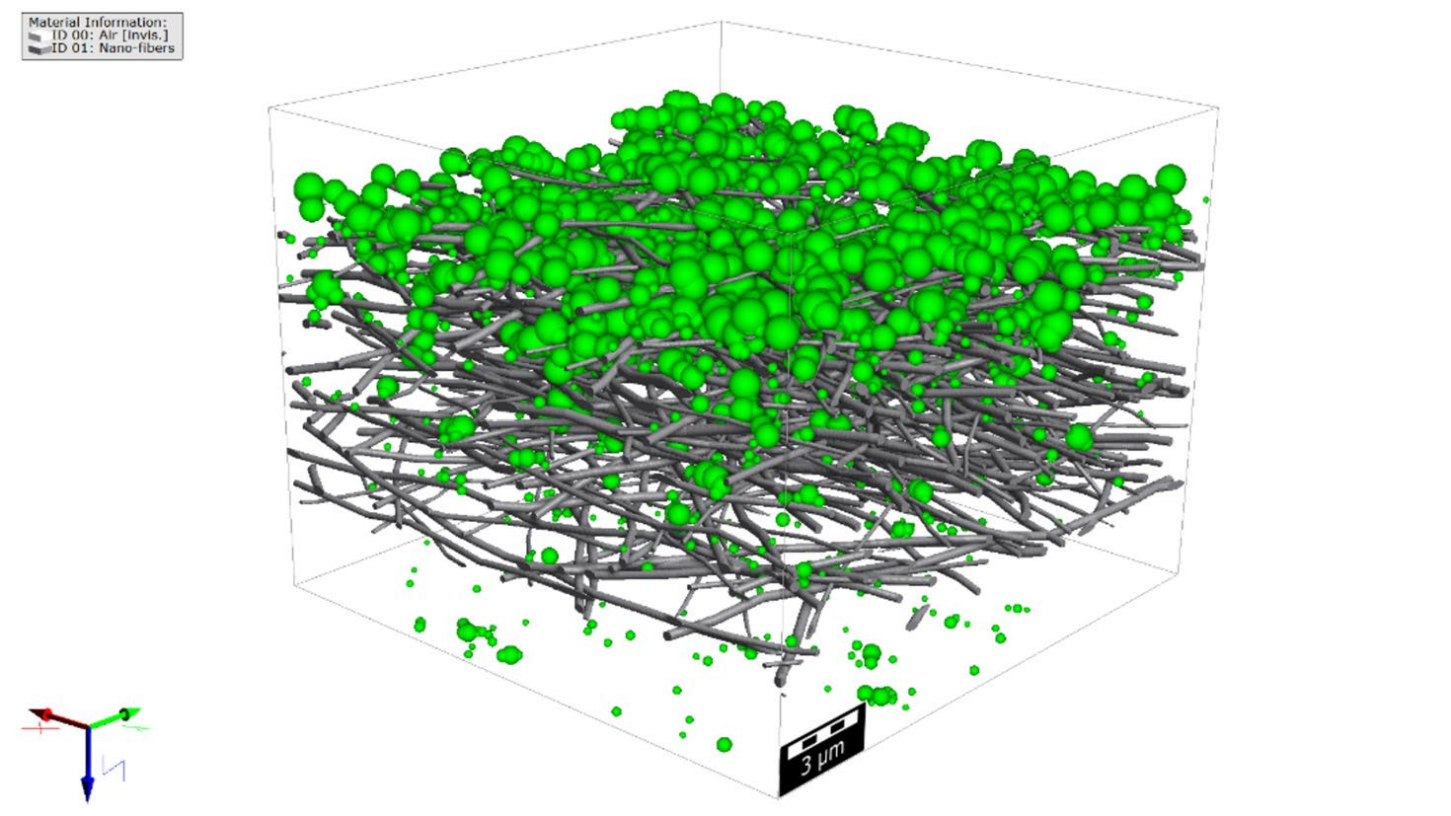
Simulated aerosols (visualized aerosols in the range of up to 1 µm) through and on the filter medium sample A.

**Figure 16:**
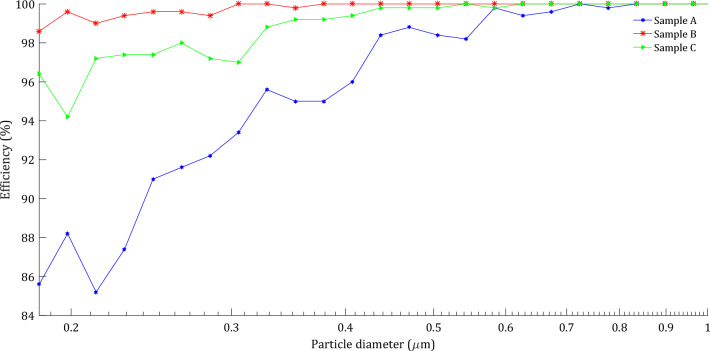
Filtration efficiency versus aerosol diameter size for filter media samples A, B, and C.

The overall and fractional efficiencies of the filter media were computed. For sample A, the total filtration efficiency by count is computed as 94.5%, and the total filtration efficiency by weight is computed as 99.3%. The initial pressure drop of the clean filter media, considering the mean flow velocity of 5.30 cm/s is computed as 53.7 Pa. The aerosols filtered through and on top of sample A are shown in Fig. 15. For better visualization of depth filtration, here only aerosols in the range of up to 1 µm are shown. The filter medium in this case had a filtration efficiency of 100% for the aerosols bigger than 0.72 µm and therefore, they will all be filtered on top of the media. In Fig. 16, the filtration efficiency by count for samples A, B, and C as a function of aerosol diameter size is presented. The initial pressure drop and overall filtration efficiency by count and weight for the three filter media samples are reported in Table 3. The results show that sample A has the lowest pressure drop and filtration efficiency which is attributed to its lower thickness.

**Table 3:** Initial pressure drop, total filtration efficiencies by count and by weight of filter media samples.

Sample	Initial pressure drop	Overall efficiency by count	Overall efficiency by weight
	(Pa)	(%)	(%)
A	53.7	94.5	99.3
B	136.6	99.8	100
C	96.8	98.5	99.8

## Conclusions

In this study, it was shown how SBC can be implemented numerically for the voxelized structure of nanofibrous filter media, for which the slip flow effects are very significant. To this end, the expression of the slip velocity was reformulated for a locally quadratic velocity profile and re-implemented in the software flow solver. This approach enables the direct simulation of the slip flow and leads to precise computation of flow and filtration through nanofibrous media. The Planar Poiseuille and Couette-Poiseuille flows were employed to validate the simulations by comparing them with the analytical solutions. Nanofibrous filter media were produced at two different concentrations and durations. The morphological analysis of nanofiber media was performed using the FE-SEM images. The fiber orientation and diameter distributions of samples were measured. This information, along with the measured porosity and thickness, was employed to model the 3D virtual nanofibrous structure of filter media. The influence of the slip length boundary condition was investigated by the proposed algorithm and the results were compared and validated with the experimentally measured air permeability values. The results pointed to excellent agreement between the simulations and experimental data. Moreover, filtration simulations considering DEHS aerosols injected with airflow through the nanofibrous filter media were conducted by solving *Stokes* equations. Initial pressure drop, fractional filtration efficiency, and overall filtration efficiency by count and weight were assessed.

## Data Availability

The data is available from the corresponding author (Dr. Parham Soltani) upon request.
